# An Update on Tobacco Control Initiatives in Comprehensive Cancer Control Plans

**DOI:** 10.5888/pcd10.120331

**Published:** 2013-06-27

**Authors:** Katherine Dunne, Susan Henderson, Sherri L. Stewart, Angela Moore, Nikki S. Hayes, Jerelyn Jordan, J. Michael Underwood

**Affiliations:** Katherine Dunne, Beth Israel Deaconess Medical Center, Boston, Massachusetts; Susan Henderson, Emory University, Atlanta, Georgia; Sherri L. Stewart, Angela Moore, Nikki S. Hayes, Jerelyn Jordan, Centers for Disease Control and Prevention, Atlanta, Georgia.

## Abstract

**Introduction:**

Comprehensive cancer control (CCC) coalitions address tobacco use, the leading cause of preventable death in the United States, through formal plans to guide tobacco control activities and other cancer prevention strategies. *Best Practices for Comprehensive Tobacco Control Programs (Best Practices)* and *The Guide to Community Preventive Services* *(The Community Guide)* are used to assist with this effort. We examined CCC plans to determine the extent to which they followed the Centers for Disease Control and Prevention’s (CDC’s) tobacco control and funding recommendations.

**Methods:**

We obtained 69 CCC plans, current as of August 1, 2011, to determine which CDC recommendations from *Best Practices* and *The Community Guide* were incorporated. Data were abstracted through a content review and key word search and then summarized across the plans with dichotomous indicators. Additionally, we analyzed plans for inclusion of tobacco control funding goals and strategies.

**Results:**

CCC plans incorporated a mean 4.5 (standard deviation [SD], 2.1) of 5 recommendations from *Best Practices* and 5.2 (SD, 0.9) of 10 recommendations from *The Community Guide*. Two-thirds of plans (66.7%) addressed funding for tobacco control as a strategy or action item; 47.8% of those plans (31.9% of total) defined a specific, measurable funding goal.

**Conclusion:**

Although most CCC plans follow CDC-recommended tobacco control recommendations and funding levels, not all recommendations are addressed by every plan and certain recommendations are addressed in varying numbers of plans. Clearer prioritization of tobacco control recommendations by CDC may improve the extent to which they are followed and therefore maximize their public health benefit.

## Introduction

Tobacco use, the leading preventable cause of death in the United States, is associated with 10 types of cancer and approximately one-third of all cancer deaths (1–4). Approximately 500,000 cancer diagnoses associated with tobacco use are made each year (4). Comprehensive tobacco control programs have been shown to effectively reduce tobacco use and therefore assist with managing the health and economic costs of smoking (3,5,6). Evidence-based tobacco control recommendations are published on a national level in 2 guidance documents, *The Guide to Community Preventive Services *(7) *(The* *Community Guide*) and *Best Practices for Comprehensive Tobacco Control Programs* (8) (*Best Practices*). *The Community Guide* is developed by independent public health and prevention experts in collaboration with the Centers for Disease Control and Prevention (CDC). It describes recommended community-based health promotion and disease prevention interventions, which are continuously updated based on scientific evidence. *Best Practices* is produced by CDC’s Office on Smoking and Health and outlines a programmatic structure for implementing interventions proven to be effective, including those described in *The Community Guide*. *Best Practices* lists 5 overarching program components and advises that individual components must work together to produce the synergistic effects of a comprehensive tobacco control program. *Best Practices* also provides state-specific funding recommendations for each component.

Comprehensive cancer control (CCC) coalitions in states, territories, and tribes publish tobacco control strategies in CCC plans. These plans are developed and implemented through the support of the National Comprehensive Cancer Control Program (NCCCP), a public health initiative funded by CDC (9,10). Evidence-based prevention has always been a focus of CCC plans, and tobacco use has long been an area addressed in many plans. In 2007, a report examining CCC plans from 38 states and the District of Columbia found “room for improvement” in the extent to which they followed CDC-recommended tobacco control strategies and funding levels (11). Since the publication of that report, national attention on tobacco control has intensified. Approximately $225 million of American Recovery and Reinvestment Act (ARRA) funding has been devoted to tobacco control. In 2010, the NCCCP released a set of 6 priorities for CCC coalitions to follow as a guide in cancer planning, one of which addresses primary prevention, specifically through tobacco control (12). More recently, CDC launched Tips from Former Smokers, a media campaign intended to educate the public about health hazards due to smoking (http://www.cdc.gov/tobacco/campaign/tips/), resulting in a doubling of calls to the national quit line (13). Because of these recent changes, we undertook a reevaluation of CCC plans to determine the extent to which more recent CCC plans followed tobacco control strategies and funding recommendations published by CDC. We expanded our analysis to include all 50 states, the District of Columbia, and all US territories and tribal jurisdictions that had CCC plans. Because the NCCCP has now been in existence for more than a decade (9), we aimed to determine how well the program currently addresses tobacco control and which areas, if any, should be addressed to better equip states, territories, and tribes to minimize cancer burden through tobacco control.

## Methods

### Data source

We obtained the most recent CCC plans available as of August 1, 2011, by searching Cancer Control P.L.A.N.E.T. (14), an online portal designed to assist in planning, implementing, and evaluating evidence-based cancer control programs. Cancer Control P.L.A.N.E.T. is sponsored by multiple organizations, including CDC, the Agency for Healthcare Research and Quality, American Cancer Society, Commission on Cancer, National Cancer Institute, and Substance Abuse and Mental Health Services Administration. Additionally, we requested plans from individual CCC program consultants within CDC’s Division of Cancer Prevention and Control, as necessary. We used the most recent plan from all 69 CCC programs located in states, the District of Columbia, territories, and tribal jurisdictions. At the time of this analysis, 47 of 69 plans (68.1%) were current, meaning that they included the year 2011; the remaining plans were in the process of being updated. All available plans were included in our analysis.

We abstracted content related to tobacco control from each plan through a review of all sections that addressed tobacco use and regulations. Sections of interest included executive summaries and other sections presumed to include specific references to tobacco control, including those with subheadings related to tobacco, funding, lung cancer, and oral cancer. We supplemented our content review with a keyword search. For example, to identify content relevant to *The* *Community Guide* recommendation on an increase in the unit price of tobacco products, the keywords *tax*, *cost*, and *price* were located in each document by using the search function. We conducted all methodological procedures similarly to those reported in the 2007 report on tobacco control in CCC plans (11).

### Coding and analysis

After data abstraction, we applied dichotomous indicators (yes or no) to each of the 15 tobacco control recommendations from *Best Practices* and *The Community Guide.* We coded recommendations as yes if all of the following conditions applied: 1) at least 1 element of the recommendation as listed in its guidance document was addressed in the plan, 2) the recommendation was listed as a current strategy/objective or could be considered ongoing (eg, a tobacco tax or ban), and 3) the recommendation related specifically to tobacco. An exception to the third criterion was the “surveillance and evaluation” recommendation from *Best Practices*, for which data were also coded as yes if they referred to general cancer surveillance or plan evaluation.

In addition to searching for CDC-recommended tobacco control strategies, we assessed whether plans addressed funding for tobacco control and whether they defined a specific, measurable funding goal. We defined a specific, measurable goal as a specific dollar amount, a percentage, or a reference to CDC-recommended funding levels. For example, New York was coded as addressing a specific funding goal because it aimed to “fund the tobacco control program interventions in proportion to CDC recommendations or greater.”

We summarized data related to tobacco control recommendations and funding levels across all 69 plans by using the above coding scheme. We directly compared our results with the 2007 analysis (11) for 6 of 10 *Community Guide* recommendations and 4 out of 5 *Best Practices* recommendations by using the χ^2 ^test of homogeneity; because of changes in tobacco control recommendations since the 2007 analysis, we were not able to directly compare all results with the 2007 analysis. Statistical tests were conducted at a .05 significance level by using SAS (SAS Institute Inc, Cary, North Carolina) version 9.2.

## Results

All 69 CCC plans incorporated at least 1 CDC-recommended, evidenced-based tobacco control strategy. Plans incorporated a mean 4.5 (standard deviation [SD], 2.1) of 5 recommendations from *Best Practices*; most plans (71.0%) addressed all 5 recommendations (Figure). Plans incorporated a mean 5.2 (SD, 0.9) of 10 *Community Guide* recommendations; nearly half (46.3%) incorporated 5 or 6 *Community Guide* recommendations, and 3 (4.3%) plans addressed none (Figure).

**Figure:**
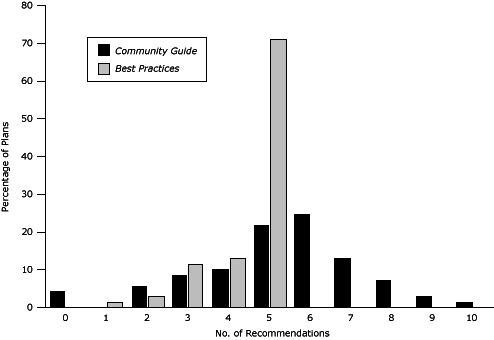
Percentage of comprehensive cancer control plans incorporating the 10 recommendations from *The Guide to Community Preventive Services (The Community Guide)* and the 5 recommendations from *Best Practices for Comprehensive Tobacco Control Programs (Best Practices)*. **Table:** 

No. of Recommendations	% of Plans Incorporating Recommendations	
	*The Community Guide*	*Best Practices*
0	4.3	0
1	0	1.4
2	5.8	2.9
3	8.7	11.6
4	10.1	13.0
5	21.7	71.0
6	24.6	Does not apply; *Best Practices* has only 5 recommendations
7	13.0	
8	7.2	
9	2.9	
10	1.4

The most commonly incorporated recommendations were “state and community programs” and “surveillance and evaluation,” both from *Best Practices* and both incorporated in 67 out of 69 plans (Table). “State and community interventions” were defined in the *Best Practices* document as comprehensive statewide tobacco control programs that combined and coordinated community-based interventions. “Surveillance and evaluation” was defined as a system to monitor and document short-term, intermediate, and long-term intervention outcomes in the population to inform policy and ensure fiscal accountability. The most common *Community Guide* recommendation incorporated was smoking bans and restrictions, incorporated in 60 of 69 plans. Elements of this recommendation as defined by *The Community Guide* included policies, regulations, and laws from private groups, nongovernmental groups, and governmental groups. As an example of a plan that incorporated the recommendation on smoking bans and restrictions, the Arizona CCC document included a plan to “advocate for enactment of a law to prohibit smoking in all enclosed public areas and workplaces (including restaurants, malls, office buildings).”

**Table:** Comparison of CDC Tobacco Control Recommendations Applied in Comprehensive Cancer Control Plans, 2007 and 2012

Recommendation	Plans Applying Recommendation		
	2007 Analysis^a^ (n = 39)	Current Analysis^b^ (n = 69)	*P* Value^c^
***Best Practices for Comprehensive Tobacco Control Programs *(** 8 **)**			
State and community programs^d^	—	97% (67)	—
Health communications interventions^e^	63%	83% (57)	.02
Cessation programs	87%	88% (61)	.81
Surveillance and evaluation	53%	97% (67)	<.001
Administration and management	40%	84% (58)	<.001
***The Guide to Community Preventive Services:* Tobacco Use Prevention and Control (** 7 **)**			
Smoking bans and restrictions	84%	87% (60)	.69
Increase the unit price of tobacco products	47%	72% (50)	.009
Mass media campaigns when combined with other interventions	61%	81% (56)	.02
Provider reminders when used alone^f^	—	19% (13)	—
Provider reminders with provider education	71%	19% (13)	<.001
Reducing client out-of-pocket costs for cessation therapies	71%	49% (34)	.03
Multicomponent interventions that include client telephone support	50%	65% (45)	.12
Community mobilization with additional interventions^g^	—	59% (41)	—
Smoke-free policies to reduce tobacco use^g^	—	70% (48)	—
Incentives and competitions to increase smoking cessation when combined with additional interventions^g^	—	3% (2)	—

Abbreviations: CDC, Centers for Disease Control and Prevention; —, comparison of data for 2007 and 2012 not possible because of changes in tobacco control recommendations since 2007.

a Based on a previous analysis of 39 comprehensive cancer control plans; the previous analysis reported only percentage of plans, not number of plans (11).

b Number of plans provided in parentheses.

c *P* value derived from χ^2^ test of homogeneity; *P* <.05 considered significant.

d State and community programs comprised separate recommendations in 2007 (11).

e Referred to as “counter marketing” in 1999 recommendations and 2007 analysis (11).

f “Provider reminders when used alone” was not included in previous analysis (11).

g These 3 recommendations were added after previous analysis (11).

Compared with 2007, CCC plans increased incorporation of all *Best Practices* strategies (Table). We found significant increases in plan adoption of best practices for surveillance and evaluation and administration and management (*P* < .001); nearly all plans (97%) incorporated the former. The percentage of plans incorporating cessation programs slightly increased from 2007 (87%) to 2012 (88%). Another *Best Practices* recommendation, new since 2007, pertaining to state and community programs, was incorporated in 97% of CCC plans.

CCC plan incorporation of *Community Guide* recommendations was more variable. The percentage of plans that incorporated recommendations to increase the unit price of tobacco products increased significantly from 47% in 2007 to 72% in the current analysis (*P* = .009), and percentage incorporating recommendations on mass media campaigns increased from 61% to 81% (*P* = .02). The percentage of plans incorporating recommendations on smoking bans and restrictions did not change, whereas the percentage incorporating provider reminders with education and reducing client costs for cessation therapies significantly decreased. Most plans incorporated recommendations added since 2007 on community mobilization with additional interventions (59%) and smoke-free policies to reduce tobacco use (70%).

A greater proportion of CCC plans addressed tobacco control funding in the current analysis than in the 2007 report. Two-thirds (66.7%) of CCC plans addressed funding in the current analysis as a strategy or action item; nearly one-third (31.9%) addressed a specific, measurable funding goal. In the previous analysis, 48.7% of plans addressed funding, and 25.6% addressed a specific goal. 

## Discussion

CCC plans incorporated nearly all *Best Practices* recommendations and approximately half of *Community Guide* recommendations. Two-thirds of plans addressed funding for tobacco control, and nearly one-third outlined a specific funding goal. Compared with a 2007 analysis, current CCC plans incorporated a larger proportion of *Best Practices* recommendations and a smaller proportion of *Community Guide* recommendations. The decrease in the proportion of *Community Guide* recommendations incorporated into CCC plans may be explained by the addition of 3 recommendations since the prior analysis. Compared with 2007, a greater proportion of plans addressed tobacco control funding and delineated a specific funding goal. The observed increase in adherence to evidence-based tobacco control recommendations and funding levels is encouraging because comprehensive, evidence-based tobacco control programs reduce smoking, and greater investment in such programs leads to greater reductions in smoking rates.

*The Community Guide* contains a dynamic set of tobacco control recommendations, whereas *Best Practices* outlines the programmatic framework and funding levels for their implementation. The 2 guidance documents are complementary and are intended to be used by states, tribes, and territories in a combination that is appropriate for each infrastructure and population. The degree to which CCC plans incorporate *Community Guide* recommendations reflects the degree to which they follow specific tobacco control strategies, whereas CCC plan incorporation of *Best Practices* recommendations may reflect broader adherence to CDC-recommended programmatic structure.

The increase in the percentage of plans addressing funding goals is an important development because there is evidence of an inverse relationship between investment in tobacco control programs and smoking prevalence (15,16). For tobacco control programs that do meet recommended funding levels, prioritizing tobacco control strategies in terms of public health impact and cost-effectiveness would assist CCC coalitions in maximizing public health benefit despite limited resources. Because there is evidence that certain interventions, such as tobacco taxation, are more effective than others in reducing smoking prevalence (17), such prioritization could prove useful in guiding the allocation of limited financial resources for cancer control coalitions.

Tobacco control contributed to an overall decline in cancer mortality in the United States between 1990 and 2000 (18), but efforts must continue to eliminate disparities across demographic groups and areas of the country. Among women, lung and bronchus cancer mortality increased between 1990 and 2000, followed by a stabilization in 2005 (19). Lung cancer death rates among women did not begin to decrease until 2007, more than a decade after the first decreases were seen among men (20). This delayed decrease was likely due to divergent smoking patterns, in that increases in smoking among women continued after men began decreasing use (4). Recent data suggest that the prevalence of cigarette smoking is declining overall among adults; however, a substantial percentage (19.3%) of US adults are current cigarette smokers (21). Additionally, 23.9% of high school students are current tobacco users, and the overall prevalence of tobacco use among high school and middle school students has not declined in recent years (22). Furthermore, cancer-specific analyses have shown that smoking prevalence persists even among those who have been diagnosed with cancer (23). Therefore, the inclusion of tobacco initiatives and goals in CCC plans should remain a priority for the NCCCP, and adherence to best practice programmatic structure is needed to help further reduce the cancer burden in all populations.

A strength of our study is the inclusion of data from all available state, territory, and tribal CCC plans. Another strength is that the study examines 2 types of recommendations: 1 type for program structure and funding and another type for interventions to be undertaken at the community level. Limitations include use of dichotomous indicators, which may not have provided a completely accurate picture of tobacco control in CCC plans. Changes in tobacco control recommendations during the last several years limited our ability to directly compare current plans and those analyzed in the previous study. Also, by considering only information included in CCC plan documents, we may have missed ongoing interventions that were omitted from plans because of space, novelty, or other considerations. In general, CCC plans include the overarching goals, objectives, and interventions that CCC coalitions hope to implement. The actual implementation of the plan and the selection of evidence-based strategies to achieve objectives are better represented in action plans and reports submitted to CDC as part of the NCCCP cooperative agreement reporting requirements, which are not publicly available. Another challenge inherent to this type of analysis is the identification of relevant content in plans that vary widely in format. CCC plans are intended to be data-driven, documented strategies that address the entire cancer control continuum by using CDC-recommended goals and objectives. CDC published an assessment tool for CCC programs to determine whether plans have clear goals, objectives, and recommended strategies (24). Researchers conducted an in-depth assessment of 66 published plans and found that most programs provided relatively comprehensive descriptions of goals, objectives, and strategies. This type of guidance may improve the usability of CCC plans for evaluation of tobacco and other prevention and control initiatives.

Our analysis shows the potential for improvements in the dissemination of tobacco control recommendations. CDC does not currently provide guidance on the prioritization of specific tobacco control recommendations; however, the use of *The Community Guide* recommendations may maximize limited resources and the potential impact of tobacco control efforts. Our study also demonstrates the need for development of resources that more specifically address the implementation, progress, and successes of CCC programs. A chronic disease management information system is in development stages; this system may serve as a platform for assessing tobacco implementation activities and outcomes for CDC and its grantees.
